# Frailty as a predictor of neurosurgical outcomes in brain tumor patients: A systematic review and meta-analysis

**DOI:** 10.3389/fpsyt.2023.1126123

**Published:** 2023-02-17

**Authors:** Jinfeng Zhu, Xichenhui Qiu, Cuiling Ji, Fang Wang, An Tao, Lu Chen

**Affiliations:** ^1^Nanjing Drum Tower Hospital, The Affiliated Hospital of Nanjing University Medical School, Nanjing, Jiangsu, China; ^2^Medical School of Nanjing University, Nanjing, Jiangsu, China; ^3^Health Science Center, Shenzhen University, Shenzhen, China; ^4^The Nethersole School of Nursing, The Chinese University of Hong Kong, Shatin, Hong Kong SAR, China

**Keywords:** brain tumor, frailty, neurosurgical outcomes, systematic review, mortality, postoperative complications

## Abstract

**Background:**

Patients with frailty are at a high risk of poor health outcomes, and frailty has been explored as a predictor of adverse events, such as perioperative complications, readmissions, falls, disability, and mortality in the neurosurgical literature. However, the precise relationship between frailty and neurosurgical outcomes in patients with brain tumor has not been established, and thus evidence-based advancements in neurosurgical management. The objectives of this study are to describe existing evidence and conduct the first systematic review and meta-analysis of the relationship between frailty and neurosurgical outcomes among brain tumor patients.

**Methods:**

Seven English databases and four Chinese databases were searched to identify neurosurgical outcomes and the prevalence of frailty among patients with a brain tumor, with no restrictions on the publication period. According to the Joanna Briggs Institute (JBI) Manual for Evidence Synthesis and the Preferred Reporting Items for Systematic reviews and Meta-Analysis (PRISMA) guidelines, two independent reviewers employed the Newcastle–Ottawa scale in cohort studies and JBI Critical Appraisal Checklist for Cross-sectional Studies to evaluate the methodological quality of each study. Then random-effects or fixed-effects meta-analysis was used in combining odds ratio (OR) or hazard ratio (RR) for the categorical data and continuous data of neurosurgical outcomes. The primary outcomes are mortality and postoperative complications, and secondary outcomes include readmission, discharge disposition, length of stay (LOS), and hospitalization costs.

**Results:**

A total of 13 papers were included in the systematic review, and the prevalence of frailty ranged from 1.48 to 57%. Frailty was significantly associated with increased risk of mortality (OR = 1.63; CI = 1.33–1.98; *p* < 0.001), postoperative complications (OR = 1.48; CI = 1.40–1.55; *p* < 0.001; *I*^2^ = 33%), nonroutine discharge disposition to a facility other than home (OR = 1.72; CI = 1.41–2.11; *p* < 0.001), prolonged LOS (OR = 1.25; CI = 1.09–1.43; *p* = 0.001), and high hospitalization costs among brain tumor patients. However, frailty was not independently associated with readmission (OR = 0.99; CI = 0.96–1.03; *p* = 0.74).

**Conclusion:**

Frailty is an independent predictor of mortality, postoperative complications, nonroutine discharge disposition, LOS, and hospitalization costs among brain tumor patients. In addition, frailty plays a significant potential role in risk stratification, preoperative shared decision making, and perioperative management.

**Systematic review registration:**

PROSPERO CRD42021248424

## Background

Histologically, brain tumors can be categorized as primary or metastatic tumors (1). The incidence rate of malignant brain tumors is 7.1/100,000 and that of benign tumors is 13.8/100,00 (2). Brain tumors can appear at any age and commonly occur in adults with a median age of 59 years (3). In addition, malignant brain tumors are the most common solid tumors in children, with more than 4,600 cases estimated in 2016 (2). Moreover, brain tumors rank as the second highest symptom-burden disease worldwide after lung cancer but account for only 1.4% of all cancer types (4, 5). It has been long recognized as producing a high rate of mortality and disability and usually has a poor prognosis for survival with diverse physical, cognitive, and behavioral impairments (5). The 5-year survival rate through the full age spectrum is just 34% on average, and only 6.1% in individuals over 75 years old. Especially, patients with glioblastoma are approximately 5%, and the median survival of newly diagnosed glioblastoma ranges from less than 1–3 years, with an average of 12–14 months (6, 7).

As the population ages and need for surgery increase, risk stratification tools have become critical to surgical planning such as age and frailty (8). Frailty describes a state of increased vulnerability and decreased physiological reserve that can be defined multidimensional components, including physical, psychological, and social factors (9). The new concept of patient frailty in surgery, particularly complex surgical intervention, including cranial neurosurgery that considers frailty in neurosurgical outcomes (8, 10). The prevalence of frailty in neurosurgery of patients with brain tumors has reached 57%. Patients with frailty are at a high risk of poor health outcomes, and frailty has been explored as a predictor of adverse events, such as perioperative complications, readmissions, falls, disability, and mortality in the neurosurgical literature (11, 12). However, the precise relationship between frailty and neurosurgical outcomes in patients with brain tumor has not been established, and thus evidence-based advancements in neurosurgical management.

Therefore, this study aimed to identify and systematically synthesize evidence to examine the relationships between frailty and neurosurgical outcomes in patients with brain tumor. The objectives were as follows: (1) to appraise the quality and level of certainty of available evidence and (2) to examine the relationships between frailty and neurosurgical outcomes in brain tumor patients.

## Methods

This systematic review was designed according to the guidelines of the Joanna Briggs Institute (JBI) (13) and was reported according to the Preferred Reporting Items for Systematic reviews and Meta-Analysis (PRISMA) guidelines (14) (Supplementary material 1). The review was registered to the International Prospective Register of Systematic Reviews (PROSPERO registration number CRD42021248424). We will continue to update any amendments on PROSPERO.

### Eligibility criteria

#### Study designs

Studies that provided observational data on cross-sectional, retrospective, or prospective associations between frailty and neurosurgical outcomes in patients with brain tumor were included. Duplicate studies, abstracts, conference proceedings, comments, letters, correspondences, editorials, and incomplete articles were excluded. Published in languages other than English and Chinese were also excluded.

#### Types of participants

Patients who underwent surgery because of confirmed brain tumor at any age based on international criteria and guideline definitions, including intracranial metastatic from systematic cancers, brain neoplasms, cerebral tumor, glioma, meningioma, hypophysoma, and pituitary tumor, were included.

#### Interest of context

Frailty was assessed using validated assessment instruments, such as the Johns Hopkins Adjusted Clinical Groups (JHACG) frailty-defining diagnosis indicator, Frail Index (FI), modified Frailty Index (mFI), Five-Factor Modified Frailty Index (mFI-5), and Hop-kins Frailty Score (HFS).

#### Types of outcome measures

Studies that reported any neurosurgery outcomes were included. The primary outcomes were mortality and postoperative complications, and the second outcomes were readmission, discharge disposition, length of stay (LOS), and hospitalization costs.

#### Data sources and search strategy

Seven electronic databases: Web of Science, EMBASE, CINAHL, Scopus, MEDLINE, PubMed, and the Cochrane Library and four Chinese databases: China National Knowledge Infrastructure, China Science and Technology Journal Database, Wanfang Database, and Chinese Biomedicine Literature Database were analyzed. The search was limited in English and Chinese, and no restriction on publication period was used. After the preliminary search of various databases to analyze the keywords and determine the index terms. We used a tailored search strategy on various databases to ensure that all available studies were obtained. We also tried any searching in the grey literature. Subsequently, searching was modified according to the databases and was limited by the language of publication. The search strategy was as follows: (frail OR frailty) AND (“brain neoplasms” OR “brain tumor” OR “cerebral tumor” OR glioma OR meningioma OR hypophysoma OR “pituitary tumor”). Supplementary material 2 describes the search strategy of MEDLINE and Web of Science on July 27, 2022. Titles, abstracts, and full texts were screened and examined for eligibility independently by two investigators. The reference lists of relevant articles were reviewed for additional studies. Corresponding authors were contacted when additional information was needed.

### Study selection

Following the database search, all identified studies were collected, and duplicates were removed. Two independent reviewers (JZ and FW) screened the titles and abstracts, and full articles were downloaded and read according to the inclusion and exclusion criteria for the assessment of eligibility. The documents screened and selected in each step were managed using Note Express V.3.3.0 software.

### Data extraction

JZ, FW and QX performed the data extraction following the PRISMA guidelines and data were extracted in tables independently by three authors. The methodological quality of the studies was assessed by two authors (JZ and FW), and any remaining disagreements were resolved by another author (QX). The extracted data included specific details about the first author, published year, country, design, number of patients, type of patients, age, gender, frailty assessment, study period, prevalence of frailty, covariates, and neurosurgical outcomes. The data were recorded in Microsoft Excel for analysis.

### Assessment of methodological quality

Risk of bias was assessed using the Newcastle–Ottawa Scale (NOS) (15) (Supplementary material 3) for cohort studies, and the JBI Critical Appraisal Checklist (16) (Supplementary material 4) for cross-sectional studies for the evaluation of the methodological quality of each study. The NOS uses two tools for case control and cohort studies and encompasses three quality parameters: selection, comparability, and exposure or outcome assessment. It assigns a maximum of four points for selection, two points for comparability, and three points for exposure or outcome (for a total of up to nine points). The NOS scores of seven or higher were considered high-quality studies, and scores of five to six denoted moderate quality (15). The JBI Critical Appraisal Checklist included 11 items, and each needed an answer of Yes, No, Unclear, or Not Applicable (16). Two researchers appraised the articles independently, and any disagreement was discussed until a consensus was reached.

### Data analysis

Random-effects or fixed-effects meta-analysis was used in combining odds ratio (*OR*) or hazard ratio (*RR*) for the categorical data and continuous data of neurosurgical outcomes. A random-effects model was used when high heterogeneity was detected, and a fixed-effects model was used when heterogeneity was low to moderate. Heterogeneity was statistically evaluated using Cochrane’s Q statistic and *I*^2^, and *I*^2^ values of 25%, 50%, and 75% were considered low, moderate, and high heterogeneity, respectively (17). Sensitivity analysis or subgroup analysis was performed when heterogeneity was high, and 95% confidence intervals (CI) were calculated for analysis. *p* < 0.05 was considered statistically significant. The finding was described in narrative form, and figures and tables were included when statistical pooling was not possible. All analyses were performed using Review Manager version 5.3. (The Cochrane Collaboration).

### Assessment of reporting bias

Reporting bias was explored using a funnel plot when the included studies were higher than 10. Risk of bias was assessed as visual inspection of a funnel plot constructed by plotting effect size versus SE.

### Quality of evidence

Quality assessment was conducted using the Grading of Recommendation Assessment, Development, and Evaluation system (18). Papers were ranked as high, moderate, low, or very low.

## Results

### Literature search process

**Figure 1:**
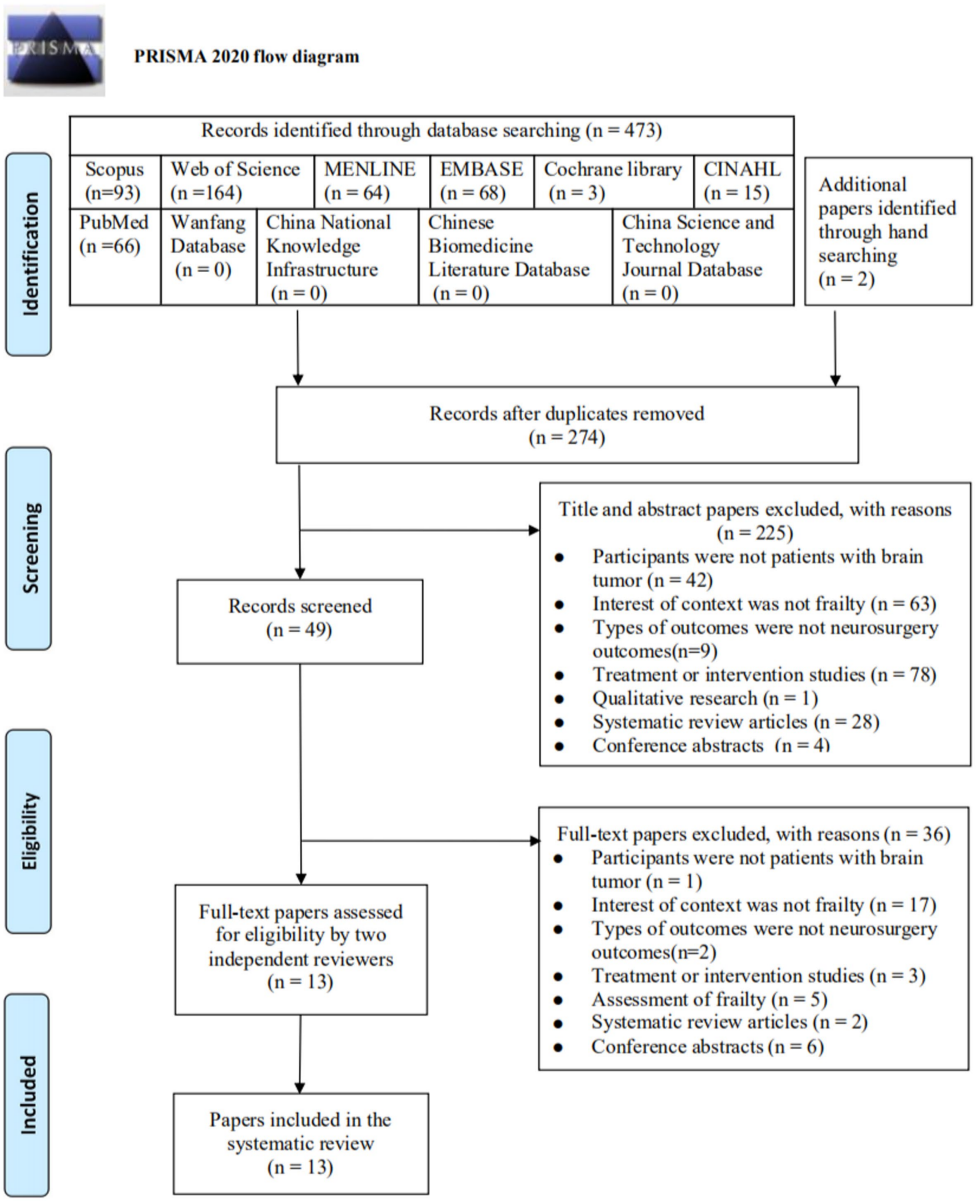
PRISMA flow diagram of literature search and study selection.

A total of 473 papers were identified through databases searching, and two papers were obtained through hand searching. After duplicates were removed, 247 were screened by reviewing the titles and abstracts, and 225 irrelevant papers were excluded. Of the 49 papers retrieved for full-text screening, 36 were excluded for the following reasons: Finally, 13 papers were included in this review. The selection process was summarized in a PRISMA flow diagram (Figure 1).

### Study characteristics

This review included 13 studies: 11 retrospective studies (19–29), one prospective study (30), and one retrospective cross-sectional study (31), with sample sizes ranging from 76 to 115,317 (Table 1). Publication locations were the United States (19–21, 23–31) and Columbia (22) between 2013 and 2021. Frailty was assessed using mFI (19, 22, 28, 29, 31), JHACG (20, 21, 26, 27), HFS (30), and mFI-5 (23–25). Prevalence of frailty ranged from 1.48% to 57%.

**Table 1:** Characteristics of included studies.

Study (year)	Country	Design	No. of patients	Type of patients	Median age (range) years	Gender (% women)	Frailty assessment	Study period	Prevalence of frailty	Covariates	Neurosurgical outcomes
Adams (19) 2013	America	Retrospective study	6,727	Inpatients who underwent operations	54.7	49.7%	mFI	The NSQIP participant use files for the period 2005 through2010	49.7%	Age, ASA, wound classification	Mortality, postoperative complications
Asemota (20) 2019	America	Retrospective study	115,317	Pituitary tumors or disorders who had undergone transsphenoidal pituitary surgery	57.14 ± 16.96 (frail) vs. 51.91 ± 15.88 (non-frail)	50.9%	JHACG	The 2000–2014 National (Nationwide) Inpatient Sample	1.48%	Age, sex, insurance type, median income quartile, race, hospital and surgery metrics	Mortality, postoperative complications, discharge dispositions, LOS, hospitalization costs,
Bonney (21) 2021	America	Retrospective study	87,835	Patients undergoing craniotomy for brain tumors	≥65:57% (frail) vs. 45.1% (non-frail)	53.0%	JHACG	The Nationwide Readmissions Database from 2010 ~ 2014	8.2%	Age, gender, insurance, and median income of the home zip code.	Mortality, In-hospital complications, discharge disposition, hospital readmission, LOS
Cloney (22) 2016	Columbia	Retrospective study	243	Geriatric patients who underwent resection of glioblastoma, including reoperation for recurrent disease.	73.1 ± 5.5	None	mFI	Columbia University Medical Center New York Presbyterian Hospital from 2000 to 2012	19.3%	Age, KPS, Charlson comorbidity score, cardiac risk	Postoperative complications, LOS
Harland (30) 2020	America	Prospective study	260	patients≥18 years old scheduled for elective resection of tumor	56.1 (frail) vs. 50.6 (non-frail)	53% (frail) vs. 41%(non-frail)	HFS	The University of Colorado over a 3-year period (October 2014 to August 2017).	25.4%	Age, race, sex, height, weight, body mass index, medical comorbidities, surgical procedure, site and side of brain tumor, brain tumor diagnosis, perioperative seizure, estimated blood loss from surgery.	Postoperative complications, discharge disposition, LOS
Huq (23) 2021	America	Retrospective cohort study	1,692	Brain tumor patients who underwent primary surgery	55.5	52%	mFI-5	At a single institution between January 1,2017 and December 31, 2018.	57%	Age, sex, race, ethnicity, ASA classification, diagnosis	Complications, 30-d readmissions, LOS, hospitalization costs,
Khalafallah (24) 2020	America	Retrospective cohort study	1,692	Adult patients who were operated on for brain tumors	55.49 ± 15.22	52.3%	mFI-5	At a single institution between January 1, 2017, and December 31, 2018	None	Age, race, ethnicity, sex, marital status	90-day postoperative mortality
Pitts (31) 2019	America	Retrospective cross-sectional study	410	Patients presenting to an academic hospital following a surgical procedure for a head and neck cancer diagnosis	61.9 ± 10.5	26%	mFI	Between January 2014 and December 2017	42.2%	Age, sex, race, BMI, oncologic stage, surgery type, smoking history, alcohol use	Mortality, perioperative complications, discharge disposition, 30-day readmission, LOS,
Sastry (25) 2020	America	Retrospective cohort study	20,333	Adult patients undergoing elective cranial surgery for tumor	54.85 ± 12.11	55.76%	mFI-5	2012–2018 NSQIP Participant Use File	41.3%	Age, gender, BMI, ASA classification, smoking status, dyspnea, significant pre-operative weight loss, chronic steroid use, bleeding disorder, tumor type, and operative time	30-day mortality, post-operative complication, discharge disposition, 30-day readmission
Shahrestani1 (26) 2020	America	Retrospective cohort study	746	Patients undergoing microscopic or endoscopic resection of a Pituitary adenomas	63.7 (frail) vs. 63.5 (non-frail)	41.6%vs. 38.3%	JHACG	The 2016 and 2017 National Readmission Database	None	Age and sex	Complications and Readmission (30-day, 90-day, 180-day), LOS, hospitalization costs
Shahrestani2 (27) 2020	America	Retrospective cohort study	13,342	Geriatric patients receiving cranial neurosurgery for a primary CNS neoplasm	73.7 ± 6.2	45.2%	JHACG	Between 2010 and 2017 by using the Nationwide Readmission Database	50.3%	Age, sex, CCI, and 10-year survival	Mortality, perioperative complications, discharge disposition, readmission, LOS, hospitalization costs
Theriault (28) 2020	America	Single-center retrospective cohort study	76	Patients who underwent intracranial meningioma resection	55.8 ± 15.3	72.6%	mFI	At Westchester Medical Center in Valhalla between August 2012 and May 2018	55.3%	Age, sex, BMI, smoking status, and tumor size (largest diameter in centimeters)	Readmission, discharge disposition, LOS
Youngerman (29) 2018	America	Retrospective cohort study	9,149	Patients who underwent neurosurgical procedures for intracranial neoplasms	<45: 22.6% 45–54:20.8% 55–64:26.5% ≥65:30.1%	52.9%	mFI	2008–2012 NSQIP Participant Use File	48.5%	Surgery category, pathology category，age, ASA class, sex, race, BMI, tobacco use, bleeding disorders, hemiplegia, ventilator dependence, sepsis, albumin level, weight loss, transfusion, corticosteroid use, chemotherapy in the past month, radiotherapy in the past 90 days, and emergency status of the case	30-day mortality, 30-day severe medical complications, 30-day severe neurologic complications, 30-day any complication, unfavorable disposition, LOS,

mFI, Modified Frailty Index; mFI-5, 5-factor Modified Frailty Index; JHACG, The Johns Hopkins Adjusted Clinical Groups; HFS, The Hop-kins Frailty Score; NSQIP, National Surgical Quality Improvement Program; ASA, American Society of Anesthesiologists; LOS, Lengths of Hospital Stay; KPS, Karnofsky Performance Status; BMI, Body Mass Index; CCI, Charlson Comorbidity Index; CNS, Central Nervous system.

### Risk of bias

A total of 12 studies (19–30) were assessed using the NOS (16), the overall studies were high quality: seven studies had scores of 9 (21, 23–28, 31), three studies had scores of 8 (19, 29, 30), and two studies had scores of 7 (20, 22) (Table 2). According to the JBI critical appraisal checklist, the methodological quality of one study (31) was strong with a score of 8.

**Table 2:** Quality assessment of studies using the Newcastle-Ottawa Scale.

Study	Year	Selection				Comparability	Outcome			Total score
		Representative of the exposed cohort	Selection of the non-exposed cohort	Ascertainment of exposure to implants	Demonstrate that outcome of interest was not present at start of study	Comparability of cohorts on the basis of design or analysis (variables)	Assessment of outcome	Was follow-up long enough for outcomes to occur	Adequacy of follow-up of cohorts	
Adams (19)	2013	1	1	1	1	1	1	0	1	8
Asemota (20)	2019	1	1	1	0	2	1	0	1	7
Bonney (21)	2021	1	1	1	1	2	1	1	1	9
Cloney (22)	2016	1	1	1	1	1	1	0	1	7
Harland (30)	2020	1	1	1	1	2	1	0	1	8
Huq (23)	2021	1	1	1	1	2	1	1	1	9
Khalafallah (24)	2020	1	1	1	1	2	1	1	1	9
Sastry (25)	2020	1	1	1	1	2	1	1	1	9
Shahrestani1 (26)	2020	1	1	1	1	2	1	1	1	9
Shahrestani2 (27)	2020	1	1	1	1	2	1	1	1	9
Theriault (28)	2020	1	1	1	1	2	1	1	1	9
Youngerman (29)	2018	1	1	1	1	2	1	0	1	8

### Frailty as a predictor of neurosurgical outcomes

#### Frailty is significantly associated with the risk of mortality in patients with brain tumor

**Figure 2:**
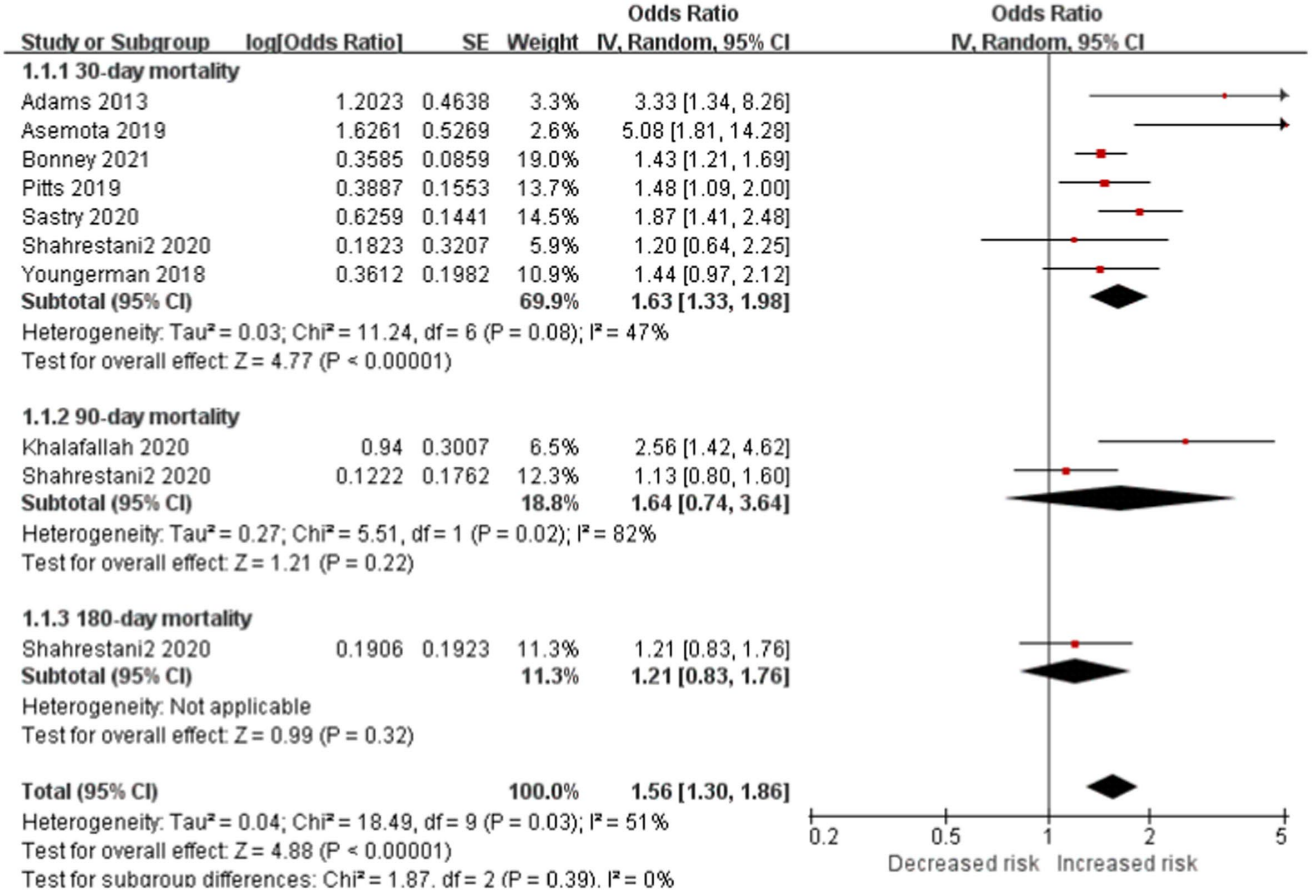
Forest plots presenting frailty is the risk of mortality in brain tumor patients.

Seven studies included 30-day mortality subgroup reported frailty is significantly associated with increased risk of mortality in patients with brain tumor (OR, 1.63; CI, 1.33–1.98; *p* < 0.0001; *I*^2^ = 47%). No significant difference in 60-day mortality and 90-day mortality subgroup between the two cohorts. However, the total outcome reported the same outcome of 30-day mortality (total OR: 1.56; CI: 1.30–1.86, *p* < 0.0001, *I*^2^ = 5 1%; Figure 2).

#### Frailty is significantly associated with the risk of postoperative complications in patients with brain tumor

**Figure 3:**
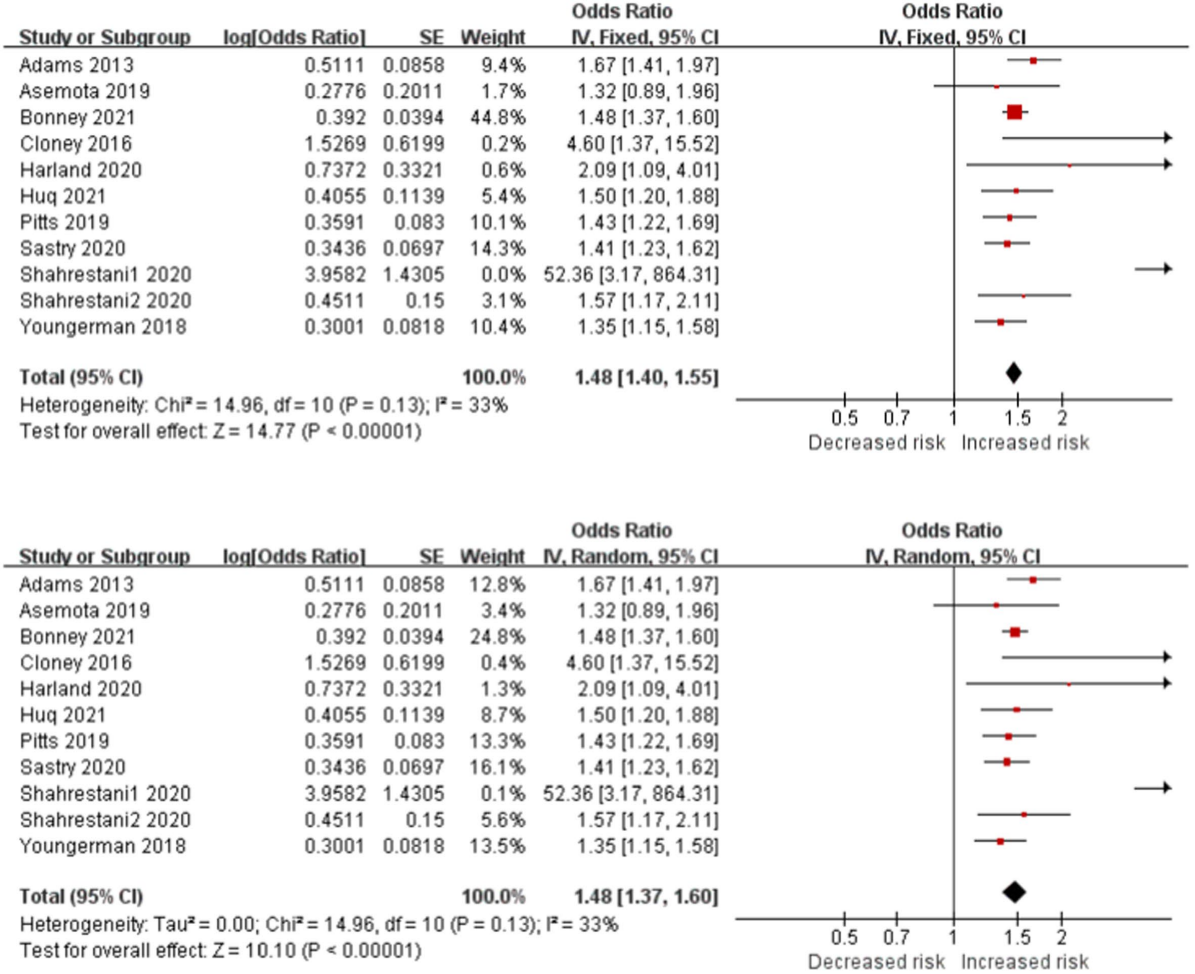
Forest plots presenting frailty is the risk of complications in brain tumor patients.

Frailty is significantly associated with increased risk of postoperative complications in patients with brain tumor in 11 studies (Figure 3). The cross meta-analysis of the fixed-effects (OR, 1.48; CI, 1.40–1.55; *p* < 0.001; *I*^2^ = 33%) and random-effects (OR, 1.48; CI, 1.37–1.60; *p* < 0.001; *I*^2^ = 33%) reported little difference between the two models, and the research results were reliable.

#### Frailty is significantly associated with the risk of nonroutine discharge position in patients with brain tumor

**Figure 4:**
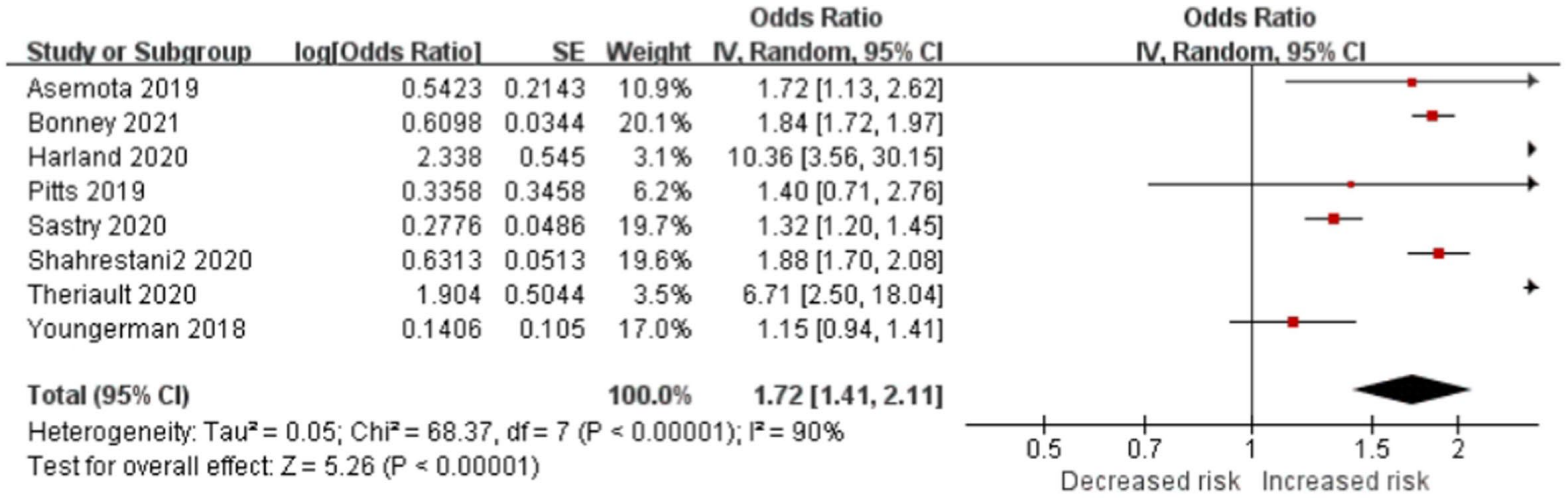
Forest plots presenting frailty is the risk of non-routine discharge disposition in brain tumor patients.

Eight studies reported discharge disposition as an outcome, and the data showed that frailty is more significantly associated with increased risk of nonroutine discharge position than home in patients with brain tumor (OR, 1.72; CI, 1.41–2.11; *p* < 0.001; *I*^2^ = 90%; Figure 4). As a result, frail patients had a higher rate of nonroutine hospital discharges compared with nonfrail patients, which encompasses transfers to skilled nursing home facilities, short-term hospitals, and home health care.

#### Frailty is significantly associated with the risk of readmission in patients with brain tumor

**Figure 5:**
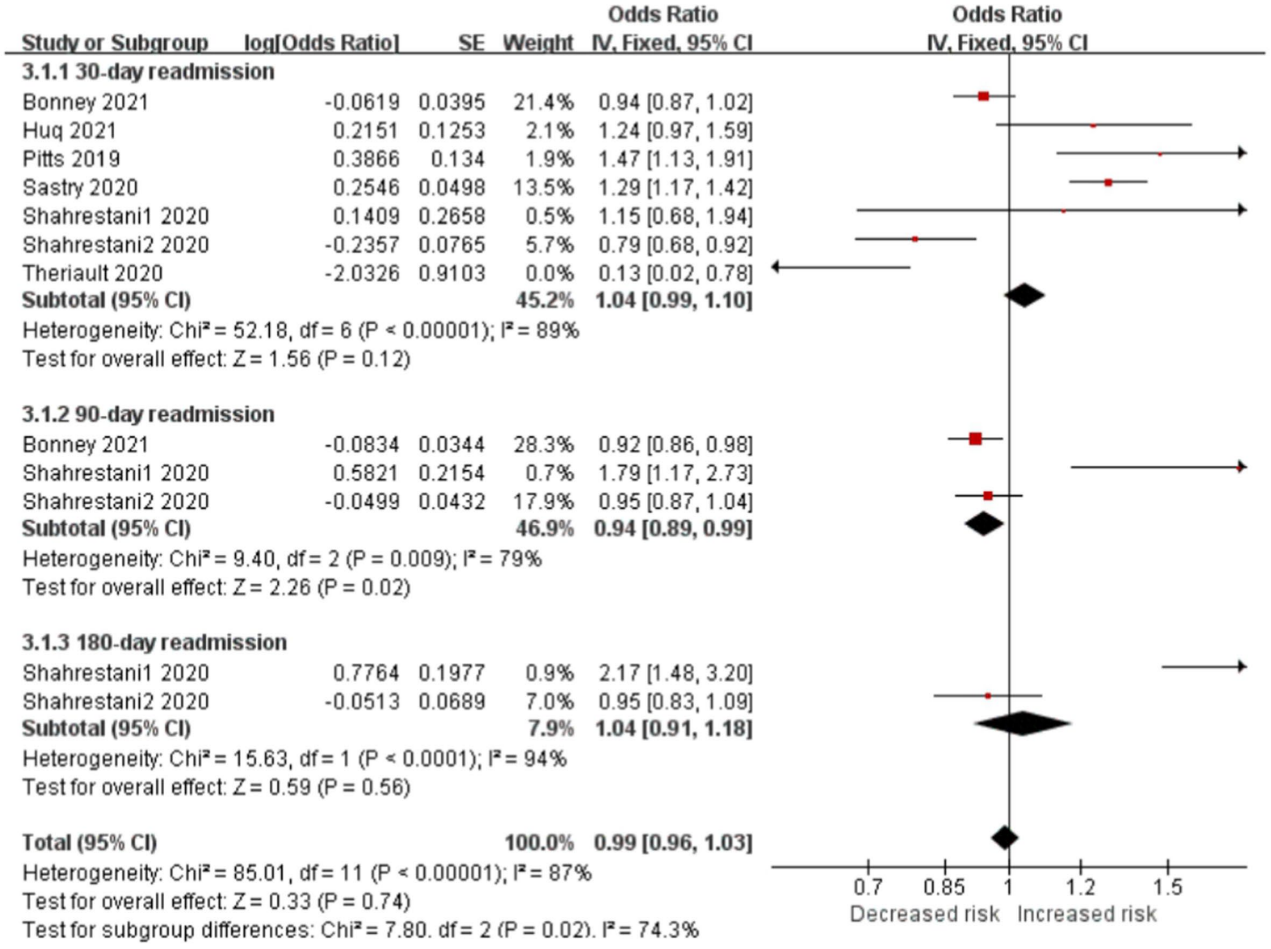
Forest plots presenting frailty is the risk of readmission in brain tumor patients.

Readmissions were classified as 30-, 90-, or 180-day subgroup. Frail patients had lower 90-day readmission rates than nonfrail patients (OR, 0.94; CI, 0.89–0.99; *p* < 0.05; *I*^2^ = 79%). However, no difference was seen at the 30-day (OR, 1.04; CI, 0.99–1.10; *p* = 0.12; *I*^2^ = 89%) or 180-day (OR, 1.04; CI, 0.91–1.18; *p* = 0.56; *I*^2^ = 9 4%) between the two cohorts. Frailty was not independently associated with readmission (OR, 0.99; CI, 0.96–1.03; *p* = 0.74; *I*^2^ = 87%; Figure 5).

#### Frailty is significantly associated with long LOS in patients with brain tumor

**Figure 6:**
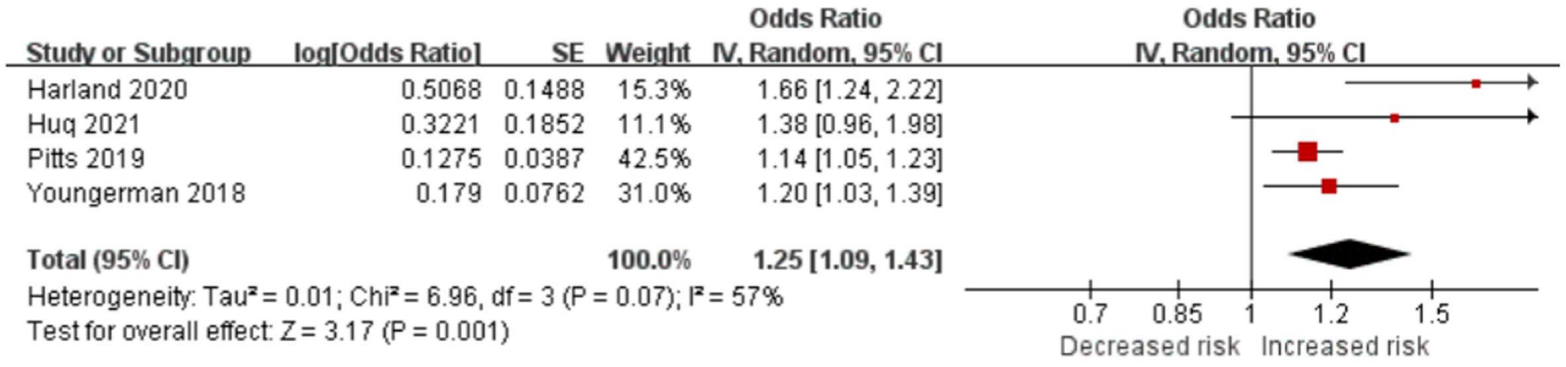
Forest plots presenting frailty is the risk of LOS in brain tumor patients.

Four studies reported frailty-prolonged LOS in patients with brain tumor (OR = 1.25; CI = 1.09–1.43; *p* = 0.001; Figure 6). Frailty significantly increased LOS in the studies by Asemota (20) (9.27 days [CI, 7.79–10.75] vs. 4.46 days [CI, 4.39–4.53], *p* < 0.001), Bonney (21) (incident rate ratio, 1.92; CI, 1.87–1.98; *p* < 0.0001), Cloney (22) (6 days vs. 4 days), Shahrestani1 (26) (13.79 ± 19.10 days vs. 4.37 ± 5.22 days, *p* < 0.001), and Shahrestani2 (27) (16.1 ± 13.9 days vs. 9.0 ± 8.1 days, *p* < 0.0001). Theriault (28) found that for every unit increase in the mFI, the expected LOS increased by 1.678 days on average, holding other variables constant (*p* = 0.046).

#### Frailty is significantly associated with higher hospitalization cost in patients with brain tumor

Frailty is significantly associated with high hospitalization costs in patients with brain tumor, as reported by Asemota (20) ($109,614.33 [*CI* $92,756.090–$126,472.50] vs. $56,370.35 [*CI* $55,595.72–$57,144.98], *p* < 0.001), Shahrestani1 (26) ($191,129.27 ± $244,619.10 vs. $89,269.91 ± $82,787.67, *p* < 0.001), and Shahrestani2 (27) ($39,114.69 ± $38,249.02 vs. $27,924.03 ± $23,886.26, *p* < 0.0001). In addition, with each one-point increase in mFI-5 score, total charges increased by $5,846 (*CI* $3,971–$7,721, *p* < 0.001) (23).

### Assessment of reporting bias

**Figure 7:**
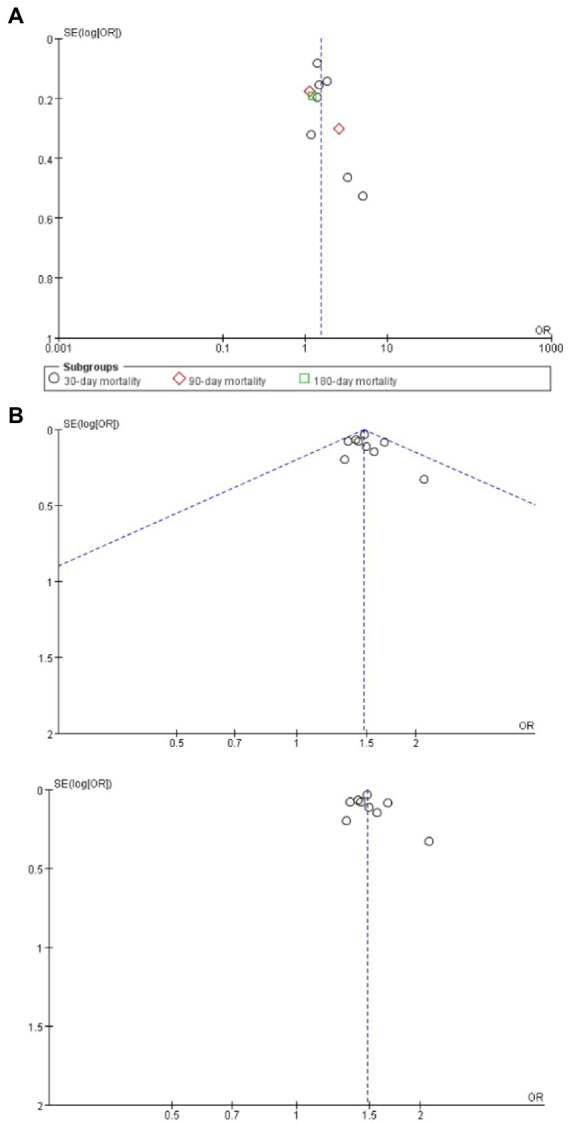
**(A)** Funnel plots assessing for report bias of frailty on mortality in brain tumor patients; **(B)** Funnel plots assessing for report bias of frailty on complications in brain tumor patients.

The effect size estimates for mortality and complications (Figure 7) all fell within the pseudo 95% confidence limits of the funnel plot. No large bias effects were reported.

## Discussion

This systematic review and meta-analysis was the first to report frailty as a predictor of neurosurgical outcomes in patients with brain tumor. Neurosurgical outcomes not only include short-term outcomes but also include long-term outcomes. Frailty was found to be an independent risk factor of neurosurgical outcomes for patients with brain tumor, and increased adverse outcomes included mortality, nonroutine discharge position rate, LOS, and hospitalization costs, especially postoperative complications. This conclusion may be particularly important not only for the elderly but also for young patients diagnosed with brain tumors. Physicians are used to thinking that age is an important predictor of complications, but frailty may be the strongest predictor. Our review found no significant difference between frail and nonfrail patients in readmission rate, particularly 30-day and 180-day readmission.

Prevalence of frailty ranged from 19.3% to 55.3% by using mFI, 41.3% to 57% by using mFI-5, 1.48% to 50.3% by using JHACG, and 25.4% by using HFS. Different assessment tools may differ slightly. One study demonstrated that the mFI is >3 times the rate of frailty compared with the JHACG method (29). Although more than 12 of methods for frailty definition have been established in the past 5 years, instrument tools that specifically target the frailty of neurosurgical patients remain limited. Furthermore, the ideal instrument of frailty defined more likely uses history and physical examination characteristics and is thus more objective according to a correlation between examination-based and diagnosis-based instruments (32). Therefore, this area warrants further exploration in the future.

The neurosurgical outcomes associated with frailty were linked to each other. For example, frailty patients have higher incidence of postoperative complications, which led to longer LOS and then increased total hospitalization costs. Additionally, postoperative medical and surgical complications result in high mortality. On the contrary, short LOS was associated with decreased hospital-acquired infections, lowered complication rates, decreased the hospitalization cost, and improved patient’s satisfaction (33, 34). These association indicated that frailty can serve as a useful risk adjustment tool related to hospital quality and reimbursement.

Owing to preoperative neurological deficits, neurosurgical oncology patients may be more heavily dependent on preoperative functional status than other surgical populations (35). Our study showed high prevalence of frailty in brain tumor patients than community-dwelling adults ranges from 9.2 to 22.7% (36), chronic heart failure whose median prevalence rate of frailty was 49.0% (37). As an independent risk factor for poor outcomes following brain tumor surgery, frailty has tremendous potential for risk stratification and outcome prediction. These allow frailty as a part of surgical risk–benefit assessment to underscore the utility of preoperative careening. Frailty should be stringently evaluated with multidisciplinary program prior to surgery, and it may aid clinical decision making (38, 39). Whether surgery or another form of management is suitable for a patient is determined (40). In addition, frailty assessment can increase intraoperative and postoperative interdisciplinary treatment program and care pathway targeting the specific elements of frailty, such as nutrition, mobilization, and hydration (41). Especially, benign brain tumor makes the majority of surgical operations exclusively elective or at least nonurgent because of the slow or nongrowing nature of these tumors. This may give us opportunity to tailor preoperative interventions or pre-habilitation to optimize surgical readiness (42) and ultimately to decrease frailty and improve postoperative outcomes.

## Limitations

Given the limitations, our study failed to include all neurosurgical outcomes, such as studies that reported that frail patients were more likely to undergo reoperations (20, 28). Further, given that most studies had a retrospective design, which included our systematic review, our analysis result may have been affected by the original study data contained in the database. Fortunately, our review included 13 studies and 257,822 patients with brain tumor. The data were obtained from a large case volume across multiple healthcare settings and countries and compensated for the limitation and improved the accuracy of the outcomes.

## Conclusion

Frailty is an independent predictor of mortality, postoperative complications, nonroutine discharge position rate, LOS, and hospitalization costs in patients with brain tumor. Frailty has a significant potential role in risk stratification, preoperative shared decision making, and perioperative management. Further study can be designed as a prospective study to explore the association between frailty and neurosurgical outcomes and quality of life.

## Contributions to the literature

- This systematic review and meta-analysis study is the first to synthesize and evaluate evidence for frailty as a predictor of neurosurgical outcomes among brain tumor patients.
- Neurosurgical outcomes include short and long-term outcomes.
- Frailty is an independent risk factor for brain tumor patients of all ages, with increased adverse outcomes, including mortality, nonroutine discharge disposition, length of stay (LOS), and hospitalization costs, especially postoperative complications. This conclusion may be important for not only elderly patients but also young patients diagnosed with a brain tumor.
- Frailty plays a significant potential role in risk stratification, preoperative shared decision making, and perioperative management.

## Data availability statement

The original contributions presented in the study are included in the article/Supplementary material, further inquiries can be directed to the corresponding author.

## Author contributions

JZ and LC: design of this systematic review protocol. JZ, FW, and XQ: literature search, data extraction and appraisal, data synthesis and interpretation, manuscript drafting. JZ, FW, AT, and CJ: data selection, data appraisal, data synthesis, manuscript critical revision, and arbitrate in cases of disagreement. All the authors have read, provided feedback, and approved the final manuscript.

## Funding

This work was supported by “Six-One Project” Top-notch Talent Research Project of Jiangsu Provincial Health and Health Commission(grant no: LGY2020012)and Shenzhen Natural Science Fund (the Stable Support Plan Program), China (grant no: 20200814144102001).

## Conflict of interest

The authors declare that the research was conducted in the absence of any commercial or financial relationships that could be construed as a potential conflict of interest.

## Publisher’s note

All claims expressed in this article are solely those of the authors and do not necessarily represent those of their affiliated organizations, or those of the publisher, the editors and the reviewers. Any product that may be evaluated in this article, or claim that may be made by its manufacturer, is not guaranteed or endorsed by the publisher.
