# Supplementary material for: Frailty as a predictor of neurosurgical outcomes in brain tumor patients: A systematic review and meta-analysis
Source: Front Psychiatry. 2023 Feb 17;14:1126123. doi: 10.3389/fpsyt.2023.1126123 (PMC9982160; doi:10.3389/fpsyt.2023.1126123)
Supplement: Supplementary file 2 [file Data_Sheet_2.PDF]

Search Strategy of Web of science on July 27,2022

# 12	164	#11 AND #3<br>Datebase= WOS, Time span = all years,Language=English
# 11	372,199	#10 OR #9 OR #8 OR #7 OR #6 OR #5 OR #4<br>Datebase= WOS, Time span = all years,Language=English
# 10	42,913	TS=(pituitary tumor*)<br>Datebase= WOS, Time span = all years,Language=English
# 9	13	TS=(hypophysoma*)<br>Datebase= WOS, Time span = all years,Language=English
# 8	28,609	TS=(meningioma*)<br>Datebase= WOS, Time span = all years,Language=English
# 7	90,990	TS=(glioma)<br>Datebase= WOS, Time span = all years,Language=English
# 6	38,063	TS=(cerebral tumor)<br>Datebase= WOS, Time span = all years,Language=English
# 5	199,824	TS=(brain tumor)<br>Datebase= WOS, Time span = all years,Language=English
# 4	201,857	TS=(brain neoplasms)<br>Datebase= WOS, Time span = all years,Language=English
# 3	38,132	#2 OR #1<br>Datebase= WOS, Time span = all years,Language=English
# 2	21,059	TS=(frail)<br>Datebase= WOS, Time span = all years,Language=English
# 1	25,189	TS=(frailty)<br>Datebase= WOS, Time span = all years,Language=English

Search Strategy of Medline on July 27,2022

# 3	64	#2 AND #1<br>Database=MEDLINE ,Time span = all years
# 2	29,992	TS=("frail" OR "frailty")<br>Database=MEDLINE ,Time span = all years
# 1	185,912	TS=<br>（"brain neoplasms" OR "brain tumor*" OR "cerebral tumor*" OR glioma* OR meningioma* OR hypophysoma* OR "pituitary tumor*")<br>Database=MEDLINE ,Time span = all years
