# Supplementary material for: Frailty as a predictor of neurosurgical outcomes in brain tumor patients: A systematic review and meta-analysis
Source: Front Psychiatry. 2023 Feb 17;14:1126123. doi: 10.3389/fpsyt.2023.1126123 (PMC9982160; doi:10.3389/fpsyt.2023.1126123)
Supplement: Supplementary file 4 [file Data_Sheet_4.PDF]

# Appendix 7.5 Critical appraisal checklist for analytical cross-sectional studies

**JBI Critical Appraisal Checklist for Analytical Cross Sectional Studies**

Reviewer________________________________________Date________________________________

-

-

Author __________________________________________Year__________Record Number ________

| | Yes | No | Unclear | Not applicable |
|---|---|---|---|---|
| 1. Were the criteria for inclusion in the sample clearly defined? | | | | |
| 1. Were the study subjects and the setting described in detail? | | | | |
| 1. Was the exposure measured in a valid and reliable way? | | | | |
| 1. Were objective, standard criteria used for measurement of the condition? | | | | |
| 1. Were confounding factors identified? | | | | |
| 1. Were strategies to deal with confounding factors stated? | | | | |
| 1. Were the outcomes measured in a valid and reliable way? | | | | |
| 1. Was appropriate statistical analysis used? | | | | |

Overall appraisal: Include Exclude Seek further info

Comments (Including reason for exclusion)

________________________________________________________________________

________________________________________________________________________

________________________________________________________________________

**Explanation of analytical cross sectional studies critical appraisal**

*How to cite:* Moola S, Munn Z, Tufanaru C, Aromataris E, Sears K, Sfetcu R, Currie M, Lisy K, Qureshi R, Mattis P, Mu P. Chapter 7: Systematic reviews of etiology and risk. In: Aromataris E, Munn Z (Editors). *JBI Manual for Evidence Synthesis.* JBI, 2020. Available from https://synthesismanual.jbi.global. https://doi.org/10.46658/JBIMES-20-08

**Analytical cross sectional studies Critical Appraisal Tool**

Answers: Yes, No, Unclear or Not/Applicable

**1. Were the criteria for inclusion in the sample clearly defined?**

The authors should provide clear inclusion and exclusion criteria that they developed prior to recruitment of the study participants. The inclusion/exclusion criteria should be specified (e.g., risk, stage of disease progression) with sufficient detail and all the necessary information critical to the study.

**2. Were the study subjects and the setting described in detail?**

The study sample should be described in sufficient detail so that other researchers can determine if it is comparable to the population of interest to them. The authors should provide a clear description of the population from which the study participants were selected or recruited, including demographics, location, and time period.

**3. Was the exposure measured in a valid and reliable way?**

The study should clearly describe the method of measurement of exposure. Assessing validity requires that a 'gold standard' is available to which the measure can be compared. The validity of exposure measurement usually relates to whether a current measure is appropriate or whether a measure of past exposure is needed.

Reliability refers to the processes included in an epidemiological study to check repeatability of measurements of the exposures. These usually include intra-observer reliability and inter-observer reliability.

**4. Were objective, standard criteria used for measurement of the condition?**

It is useful to determine if patients were included in the study based on either a specified diagnosis or definition. This is more likely to decrease the risk of bias. Characteristics are another useful approach to matching groups, and studies that did not use specified diagnostic methods or definitions should provide evidence on matching by key characteristics.

**5. Were confounding factors identified?**

Confounding has occurred where the estimated intervention exposure effect is biased by the presence of some difference between the comparison groups (apart from the exposure investigated/of interest). Typical confounders include baseline characteristics, prognostic factors, or concomitant exposures (e.g. smoking). A confounder is a difference between the comparison groups and it influences the direction of the study results. A high quality study at the level of cohort design will identify the potential confounders and measure them (where possible). This is difficult for studies where behavioral, attitudinal or lifestyle factors may impact on the results.

**6. Were strategies to deal with confounding factors stated?**

Strategies to deal with effects of confounding factors may be dealt within the study design or in data analysis. By matching or stratifying sampling of participants, effects of confounding factors can be adjusted for. When dealing with adjustment in data analysis, assess the statistics used in the study. Most will be some form of multivariate regression analysis to account for the confounding factors measured.

**7. Were the outcomes measured in a valid and reliable way?**

Read the methods section of the paper. If for e.g. lung cancer is assessed based on existing definitions or diagnostic criteria, then the answer to this question is likely to be yes. If lung cancer is assessed using observer reported, or self-reported scales, the risk of over- or under-reporting is increased, and objectivity is compromised. Importantly, determine if the measurement tools used were validated instruments as this has a significant impact on outcome assessment validity.

Having established the objectivity of the outcome measurement (e.g. lung cancer) instrument, it’s important to establish how the measurement was conducted. Were those involved in collecting data trained or educated in the use of the instrument/s? (e.g. radiographers). If there was more than one data collector, were they similar in terms of level of education, clinical or research experience, or level of responsibility in the piece of research being appraised?

**8. Was appropriate statistical analysis used?**

As with any consideration of statistical analysis, consideration should be given to whether there was a more appropriate alternate statistical method that could have been used. The methods section should be detailed enough for reviewers to identify which analytical techniques were used (in particular, regression or stratification) and how specific confounders were measured.

For studies utilizing regression analysis, it is useful to identify if the study identified which variables were included and how they related to the outcome. If stratification was the analytical approach used, were the strata of analysis defined by the specified variables? Additionally, it is also important to assess the appropriateness of the analytical strategy in terms of the assumptions associated with the approach as differing methods of analysis are based on differing assumptions about the data and how it will respond.
